# Striking Elevations in Aminotransferases in a Case of Congestive Hepatopathy Without Concurrent Hypotension

**DOI:** 10.7759/cureus.29088

**Published:** 2022-09-12

**Authors:** Kyle N Kaneko, Zacharie D Finneman, Phillip M Avila, Jayton A Lim, Suporn Sukpraprut-Braaten

**Affiliations:** 1 Internal Medicine, Unity Health, Searcy, USA; 2 Graduate Medical Education, Kansas City University, Kansas City, USA; 3 Graduate Medical Education, Unity Health, Searcy, USA

**Keywords:** shock liver, elevated aminotransferases, ischemic hepatopathy, congestive heart failure, congestive hepatopathy

## Abstract

Congestive hepatopathy results from passive venous congestion often in the setting of right heart failure. Injury to the liver due to congestion is often asymptomatic and may be difficult to recognize and diagnose. The degree of elevations in aminotransferases varies in cases of congestive hepatopathy but usually stays within two to three times the upper limit of normal. Here, we report an interesting case of congestive hepatopathy that presented with striking elevations of aminotransferases in the 2000s international units/liter a few days after admission without concurrent hypotension.

## Introduction

The liver’s high metabolic activity and complex vascularization make it susceptible to hemodynamic changes [1]. Congestive hepatopathy occurs secondary to various cardiovascular conditions that lead to persistent passive venous congestion in the liver [1]. Congestive hepatopathy often results from right heart failure but can also occur from other pathologies in the inferior vena cava or hepatic veins [2]. Common causes of this include congestive heart failure, cor pulmonale, valvulopathies, right ventricular dysfunction, constrictive pericarditis, and severe pulmonary hypertension [3]. Congestive hepatopathy is also common in patients with congenital heart disease who have had a Fontan procedure, which directs systemic venous return to the pulmonary artery, bypassing the right ventricle [3].

Diagnosis of this condition remains challenging as clinical manifestations, laboratory testing, and imaging findings are nonspecific. Patients with congestive hepatopathy usually remain asymptomatic and diagnosis may only be suspected after abnormal liver biochemical tests during routine evaluation [2]. The degree of elevation in aminotransferases varies in cases of congestive hepatopathy but typically stays within two to three times the upper limits of normal [4]. Occasionally patients may have higher levels likely due to coexisting ischemic hepatitis from decreased cardiac output [4,5]. Drastic elevations in aminotransferases exceeding 1000 international units/liter have been reported in patients with shock or hypotension due to heart failure [6]. Here, we report an interesting case of congestive hepatopathy that presented with striking elevations of aminotransferases in the 2000s international units/liter a few days after admission without concurrent hypotension or shock.

## Case presentation

A 72-year-old male presented to the emergency department with the chief complaint of increased shortness of breath, orthopnea, altered mental status, and slightly slurred speech. The patient underwent dialysis the day before presenting to the emergency department and had three liters of fluid removed. He went to the hospital due to persistent shortness of breath and increased oxygen requirement. He has a past medical history of end-stage renal disease on hemodialysis, atrial fibrillation on warfarin, right-sided heart failure with a last known ejection fraction of 54% (two years ago), hypertension, type 2 diabetes mellitus, gout, and hyperlipidemia. He denied any history of liver disease. The patient was a non-smoker, non-alcoholic, and had a noncontributory family history. His past surgical history included a right hip replacement. His home medications include allopurinol, bumetanide, lisinopril, metoprolol succinate, nifedipine, neutral protamine hagedorn insulin, pravastatin, sertraline, warfarin, and trazodone. On examination, he was tachypneic and tachycardic, but other vitals were stable. Lungs were mostly clear with mild rales in the bases bilaterally. The cardiac exam showed tachycardia with a regular rhythm and no extra heart sounds. His abdomen was soft, non-tender, and non distended. His mental status was alert and oriented to person and place, but not time. His speech was slightly slurred, but cranial nerves, muscular strength, and sensation were intact.

**Figure 1:**
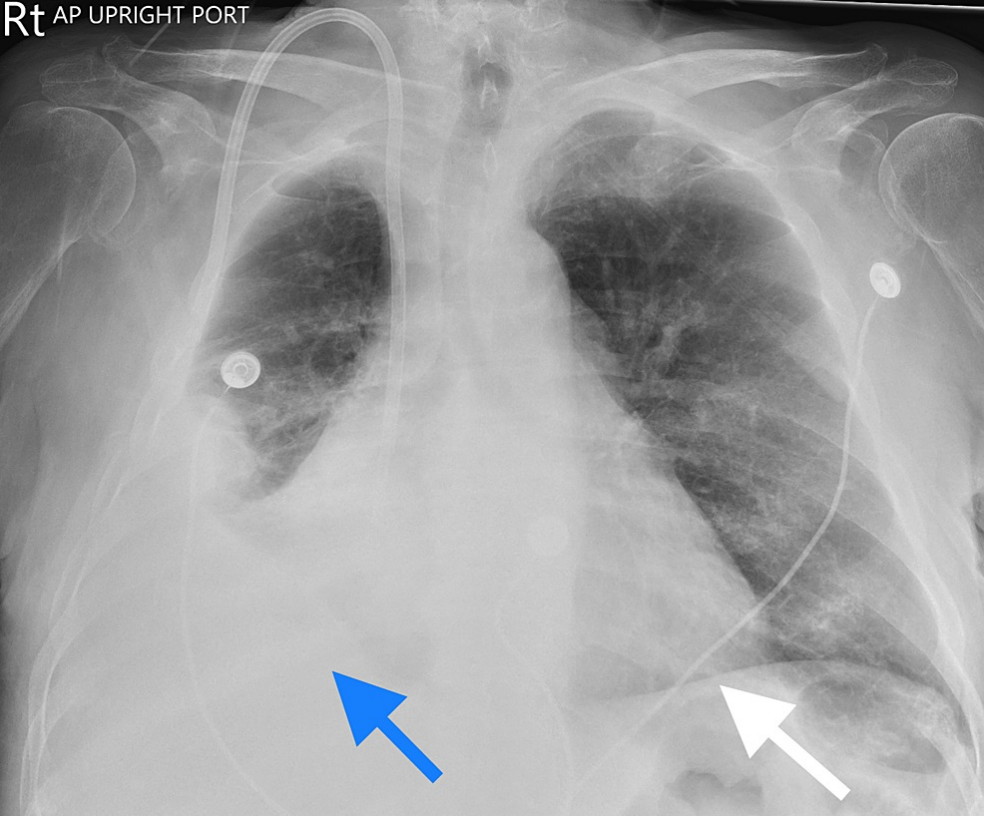
Initial Chest Radiograph Anterior-posterior chest radiograph: Blue Arrow: moderate right pleural effusion with ill-defined nodular density. White arrow: Cardiomegaly with congestive changes.

Please see Table 1 and Table 2 for the initial complete blood count and comprehensive metabolic panel. Arterial blood gas on admission showed respiratory alkalosis and metabolic acidosis (Table 3). His initial lactate level was 4.6 mmol/L. He was also noted to have a supratherapeutic international normalized ratio (INR) of 6.6 with a prothrombin time of 64.7 seconds and a partial thromboplastin time of 54.2 seconds. Brain natriuretic peptide (BNP) was 1896 pg/mL and COVID- 19 polymerase chain reaction was negative. Aminotransferases were elevated with aspartate aminotransferase (AST) and alanine aminotransferase (ALT) being 565 international units/liter and 533 international units/liter, respectively. Chest radiograph showed a moderate right pleural effusion with an ill-defined nodular density and cardiomegaly with congestive changes (Figure 1). A non-contrast computed tomography of the head was negative for bleeding or masses. The patient was given furosemide 80 milligrams, as a one-time push in the emergency department. Nephrology was consulted and urgent dialysis was done to remove volume and correct the acidosis. Intravenous furosemide of 40 milligrams per day was ordered, warfarin was withheld, an echocardiogram was ordered, cardiology was consulted, and he was admitted to the hospital.

**Table 1:** Initial Complete Blood Count

Test	Value	Reference Range
White Blood Cell Count	12.4 th/uL	4.8 – 10.8 th/uL
Red Blood Cell Count	4.50 mil/uL	4.70 – 6.10 mil/uL
Hemoglobin	13.1 g/dL	13.5 – 18.0 g/dL
Hematocrit	41.2 %	40.0 – 54.0 %
Mean Corpuscular Volume	92 fL	80 – 94 fL
Mean Corpuscular Hemoglobin	29.1 pg	27.0 – 31.0 pg
Mean Corpuscular Hemoglobin Concentration	31.8 g/dL	33.0 – 37.0 g/dL
Red Cell Distribution Width	15.9 %	11.5 – 14.5 %
Platelet Count	338 th/uL	150 – 450 th/uL
Absolute Neutrophil Count	10.7 th/uL	2.30 – 8.10 th/uL
Neutrophil Percent	86.1 %	40.0 – 80.0 %
Lymphocyte Percent	5.8 %	20.0 – 40.0 %
Monocyte Percent	7.1 %	0.0 – 10.0 %
Eosinophil Percent	0.0 %	0.0 – 5.0 %
Basophil Percent	0.2 %	0.0 – 2.0 %
Immature Granulocyte Percent	0.8 %	0.0 – 1.0 %

**Table 2:** Initial Comprehensive Metabolic Panel

Test	Value	Reference Range
Sodium	133 mmol/L	137 – 145 mmol/L
Potassium	5.2 mmol/L	3.5 – 5.0 mmol/L
Chloride	95 mmol/L	98 – 107 mmol/L
Bicarbonate	15 mmol/L	22 – 30 mmol/L
Anion Gap	23 mmol/L	5 – 15 mmol/L
Glucose	82 mg/dL	75 – 110 mg/dL
Blood Urea Nitrogen	36 mg/dL	9 – 20 mg/dL
Creatinine	3.6 mg/dL	0.8 – 1.5 mg/dL
Glomerular Filtration Rate Non-African American	16.8 mL/min	
Glomerular Filtration Rate African American	20.3 mL/min	
Calcium	9.6 mg/dL	8.4 – 10.2 mg /dL
Total Bilirubin	2.5 mg/dL	0.1 – 1.3 mg/dL
Alkaline Phosphatase	156 U/L	38 – 126 U/L
Aspartate Aminotransferase	565 U/L	5 – 34 U/L
Alanine Aminotransferase	553 U/L	11 – 55 U/L
Total Protein	8.4 g/dL	6.3 – 8.2 g/dL
Albumin	3.9 g/dL	3.2 – 5.0 g/dL

**Table 3:** Initial Arterial Blood Gas Abbreviations: pH – potential hydrogen, pCO2 – partial pressure of carbon dioxide, pO2 – partial pressure of oxygen

Test	Value	Reference Range
pH	7.40	7.35 – 7.45
pCO2	22.0 mmHg	35.0 – 45.0 mmHg
pO2	61.5 mmHg	80.0 – 90.0 mmHg
Bicarbonate	13.5 mm/L	22.0 – 26.0 mm/L
Base Excess	-9.2 mm/L	-2 – +2 mm/L
Oxygen Saturation	87.9%	95.0 – 100 %
Carboxyhemoglobin	1.3%	0.0 – 3.0 %
Methemoglobin	0.7 g/dL	0.0 – 1.5 g/dL

On the second day, his transaminitis significantly worsened with aspartate aminotransferase (AST) and alanine transaminase (ALT) increasing to 1988 international units/liter and 1705 international units/liter, respectively. A viral hepatitis panel and right upper quadrant ultrasound were ordered. Hepatology was consulted and medications were reviewed. The patient’s vitals remained stable with no episodes of hypotension. On the third day, transaminitis continued to worsen with AST and ALT increasing to 2466 international units/liter and 2352 international units/liter, respectively. Despite the drastic elevations in his aminotransferases, he denied any changes in mental status, his vitals remained stable, and his prothrombin time returned to normal limits. The viral hepatitis panel was negative, and the right upper quadrant ultrasound was normal. After a review of his medications, pravastatin was discontinued for potential hepatotoxicity and worsening of aminotransferases. His home medication allopurinol was not continued upon admission. The echocardiogram ordered on admission showed an ejection fraction of 33%, which was reduced from 54% two years ago. Echocardiogram also showed a right ventricular systolic pressure of 67.0 mmHg, indicating severe pulmonary hypertension. The consulted hepatologist concluded that the transaminitis was likely due to a combination of hepatic congestion secondary to severe pulmonary hypertension, volume overload, and ischemia from congestive heart failure. The elevations in aminotransferases were likely from the initial insult on presentation. Continued dialysis and volume removal were recommended. Over the next few days, he received his scheduled dialysis and his liver enzymes slowly returned to normal limits. 

## Discussion

Congestive hepatopathy is a pathological condition that describes liver damage often from right heart failure or a variety of cardiovascular conditions resulting in passive venous congestion in the liver [1]. Patients with this condition are usually asymptomatic and may only be suspected from abnormal liver biochemical tests on routine evaluation [2]. Symptomatic patients can present with dull right upper quadrant pain, jaundice, or ascites [7]. Splenomegaly is rare but can occur in certain patients [7]. The hepatojugular reflex is usually present and can be useful for distinguishing hepatic congestion from primary intrahepatic liver disease or Budd-Chiari syndrome [7]. In our patient, no hepatomegaly, right upper quadrant pain, ascites, or jaundice was noted. The hepatojugular reflex and jugular venous distention were not assessed.

Laboratory findings are nonspecific and of little use for diagnosis as they remain in ranges near normal until the advanced stages of the disease [8]. The most common liver biochemical abnormality is hyperbilirubinemia with total bilirubin that rarely exceeds 3 mg/dL [9]. Serum alkaline phosphatase is usually normal or slightly elevated in acute heart failure even in the presence of jaundice [7]. Alkaline phosphatase may also be elevated in the setting of chronic heart failure [7]. Serum aminotransferases may be elevated up to two to three times the normal value and may occasionally have higher levels likely due to coexisting ischemic hepatitis following decreased cardiac output [5]. Drastic elevations in aminotransferases exceeding 1000 international units/liter have been seen in patients with hypotension due to heart failure [6]. On presentation, our patient presented with a total bilirubin of 2.5 mg/dL, alkaline phosphatase of 156 international units/liter, AST of 565 international units/L, and an ALT of 554 international units/liter. Throughout our patient’s hospital course, his total bilirubin never exceeded 3 mg/dL, his alkaline phosphatase remained in the 135 - 150 international units/liter range, and no episodes of hypotension occurred. Our patients AST and ALT peaked at 2466 international units/liter and 2352 international units/liter, respectively, a few days after presentation. 

Diagnosis of hepatic congestion involves both clinical and laboratory investigations. Careful history review for risk factors for liver disease, history of liver disease, illicit drug use, alcohol use, and hepatotoxic medications need to be evaluated [9]. Laboratory testing for viral hepatitis and N-terminal-pro-BNP can help aid in the diagnosis [10]. N-terminal-pro-BNP in one study was found to distinguish heart failure-related ascites from cirrhosis with a sensitivity of 98 percent [10]. In our patient, N-terminal-pro-BNP was not ordered, but BNP was 1896 pg/mL. Electrocardiograms and echocardiography are also done to evaluate cardiac function. When evaluating a patient with congestive hepatopathy, other diagnoses to consider include viral hepatitis, autoimmune hepatitis, Wilson disease, alpha-1 antitrypsin deficiency, Budd-Chiari syndrome, and thyroid dysfunction [7,9]. Ultrasonography with Doppler studies of the right upper quadrant is warranted to rule out possible thrombosis and Budd-Chiari syndrome [7]. Characteristic ultrasound findings of congestive hepatopathy seen in the inferior vena cava and hepatic veins along with a detailed clinical history can also help confirm the diagnosis [11]. In patients with ascites, diagnostic paracentesis may aid in the diagnosis with the serum-to ascites albumin gradient being greater or equal to 1.1, demonstrating portal hypertension [12]. Improvement in aminotransferases with the treatment of the underlying cardiac condition also aids in the diagnosis [6,8]. A liver biopsy can confirm the diagnosis but is rarely required [2]. After his medications were reviewed, pravastatin was the only hepatotoxic medication found and was discontinued. Pravastatin was unlikely to be the cause of his significant transaminitis as he had taken it for many years without issues. The viral hepatitis panel and right upper quadrant ultrasound were unremarkable. An echocardiogram was ordered that demonstrated a reduced ejection fraction of 33% and a right ventricular systolic pressure of 67.0 mmHg indicating severe pulmonary hypertension. Thyroid studies were within normal limits. Other labs for autoimmune hepatitis, Wilson disease, and alpha-1 antitrypsin deficiency were not ordered due to suggestive clinical, laboratory, and imaging findings.

Management of congestive hepatopathy revolves around treating the underlying heart disease [2,9]. Hepatic congestion may respond well to diuretics, although excess diuresis should be cautioned to avoid impaired hepatic perfusion [13]. Patients may also present with an underlying coagulopathy, increasing sensitivity to warfarin anticoagulation [14]. Coagulation status should be monitored carefully along with other drugs that require hepatic metabolism. In our patient, urgent dialysis was done to remove volume overload suggested by clinical symptoms, and a chest x-ray showed cardiomegaly with congestive changes. After removing excessive volume with a few dialysis sessions, aminotransferases improved. Our patient also presented with a supratherapeutic INR of 6.7 and a prothrombin time and partial thromboplastin time of 65.6 seconds and 54.2 seconds, respectively. No active bleeding was noted, and warfarin was withheld. Coagulation labs normalized over time with scheduled dialysis. 

## Conclusions

This case presents an interesting manifestation of congestive hepatopathy with aminotransferases in the 2000s international units/liter without hypotension. The diagnosis of congestive hepatopathy is challenging as laboratory testing and clinical features vary and are nonspecific. When evaluating a patient with suspected congestive hepatopathy it is important to access cardiac function and treat the underlying heart disease. Ultrasound of the liver and hepatic vasculature may also aid in the diagnosis. More research should be conducted to determine a consistent diagnostic and treatment plan.
